# Comparing the validity of anthropometric measurements in identifying malnutrition status of older age people in Borena district, North Central Ethiopia: a cross_sectional study

**DOI:** 10.1186/s12877-022-03467-9

**Published:** 2022-10-03

**Authors:** Abdu Dawed, Tefera Chane Mekonnen, Muluken Genetu, Sisay Eshete Tadesse, Reta Dewau, Amare Muche, Aregash Abebayehu Zerga, Fanos Yeshanew Ayele, Tiffany K. Gill

**Affiliations:** 1 Dessie Comprehensive Specialized Hospital, Dessie, North Eastern Ethiopia; 2 Human Nutrition and Dietetics Department, School of Public Health, College of Medicine and Health Sciences, Wollo University, Dessie, Ethiopia; 3 Department of Health Service Management, School of Public Health, College of Medicine and Health Sciences, Wollo University, Dessie, Ethiopia; 4 Department of Epidemiology and Biostatistics, School of Public Health, College of Medicine and Health Sciences, Wollo University, Dessie, Ethiopia; 5 Adelaide Medical School, Faculty of Health and Medical Sciences, the University of Adelaide, Adelaide, South Australia Australia

**Keywords:** Anthropometric indices, Older age, Malnutrition, MNA, Reliability, Validity

## Abstract

**Background:**

Malnutrition among older age people is becoming significantly higher in spite of improvements in the health care system. Life expectancy of Ethiopian elders is increasing; but reliable and valid tools for screening and diagnosis of malnutrition in this subgroup are limited. This study aimed to assess the validity of anthropometric measurements: Mid Upper Arm Circumference (MUAC), Body Mass Index (BMI), and Calf Circumference (CC) in detecting malnutrition status of older age people in Ethiopia.

**Methods:**

A community based cross-sectional study was conducted in Borena District from January to March, 2020. A total of 421 participants aged were systematically included in the study. To test reliability and validity of the measurements,Cronbach’s α coefficient and Pearson’s correlations were used, respectively. The full Mini-Nutritional Assessment (MNA) tool was used to diagnosis malnutrition. Overall accuracy, sensitivity and specificity of BMI, MUAC and CC were estimated using Receiver Operating Characteristic curves. The Youden Index was used to determine the best cut-off point.

**Results:**

The reliability of BMI, MUAC and CC by Cronbach’s alpha was found 0.847. Significant positive correlations between MNA, BMI(*r* = 0.56, *p* < 0.01); MNA, MUAC(*r* = 0.43, *p* < 0.01; and MNA, CC(*r* = 0.52, *p* < 0.01) revealed. The area under the curve (AUC) of BMI, MUAC and CC were found: 0.98(95% CI, 0.96–0.99, *p* < 0.001), 0.94(95% CI, 0.89–0.98, *p* < 0.001) and 0.96(95% CI, 0.94–0.98, *p* < 0.001) indicating the overall accuracy respectively. The sensitivity and specificity of BMI, MUAC and CC using established cut off points were found: 90%, 96%; 78%, 94% and 84%, 95% respectively. However, using the Youden index the best cut-off point, the sensitivity and specificity of MUAC and CC were 88%, 86%; 92% and 89% respectively and adjusted for age and sex.

**Conclusions:**

The current study demonstrated that BMI was a reliable and valid method to identify the malnutrition status of older age people. A MUAC value of 19 cm and CC of 30 cm were simple and efficient cut-off points for the determination of malnutrition in the older age people. A future study is needed to validate the validity of BMI, MUAC and CC against biochemical tests as gold standard.

**Supplementary Information:**

The online version contains supplementary material available at 10.1186/s12877-022-03467-9.

## Background

As medical advances have increased the availability and access to better preventive care, people have been living longer, and ageing has imposed a global public health challenge [1, 2]. According to the Population Division of the Department of Economic and Social Affairs 2020 report, the estimated number of individuals aged 65 years or beyond will double from 727 million in 2020 to 1.5 billion in 2050, which will comprise about 16% of the global population [3]. Population ageing in developing countries is rapidly increasing as these countries have experienced greater demographic, economic and sociological changes. In Ethiopia, for example, the current life expectancy is 66.71 years, which has increased by 15.86 years since 2000 and older people comprise 4% of the general population [3, 4].

Increasing numbers of older age people in the global community require reconsideration of the suitability of health infrastructures [5]. This population group is vulnerable to malnutrition and chronic non-communicable disease. It is estimated that up to 50% of the older age population in the Ethiopian community have malnutrition, which leads to prolonged hospitalization, repeated infection, increased hospital readmissions by up to 30%, healthcare costs and mortality [6–10]. Older age populations are uniquely susceptible to malnutrition due to the association of ageing with factors that influence nutritional status such as decreased appetite, decreased energy expenditure, weight loss, taste and smell changes, feelings of loneliness and depression, difficulty chewing, fatigue and co-existing morbidities. For older age people, the potential consequences of malnutrition include a decline in functional status, impaired muscle function, decreased bone mass, immune dysfunction, anemia, reduced cognitive function, increased susceptibility to infection, and poor wound healing [10, 11].

To address the health problems among older people, provision of improved healthcare, both in hospital and in the community, from early detection to preventive care, is imperative [12]. The Mini-Nutritional Assessment (MNA) tool, which was developed in 1994, is a non-invasive, reliable and extensively evaluated nutritional assessment tool for free-living and clinically relevant older age populations [2, 13]. The full MNA has been validated in many studies, and used as a reference standard to validate other screening tools globally [14–31]. The tool has been incorporated in at least 42 Electronic Health Records Software Companies to be practiced using smartphones [32]. The tool includes body-mass index (BMI), mid-upper arm circumference (MUAC) and calf circumference (CC) together with recent nutrient intake, recent weight loss, functional mobility, recent acute disease or psychological stress and neurological problems.

Compared to the use of MNA, the assessment of nutritional status by a single or multiple anthropometry measurement is simple, non-invasive and time saving in health facilities and as part of community-based screenings of malnutrition. Anthropometry also remains the most practical tool for the assessment of nutritional status among members of the community in developing countries where scarced resources and high patient load exist, in spite of some limitations, such as do not provide uniform body composition information and recent nutritional disturbance [24]. Moreover, the use of anthropometric measurement tools as an alternative to MNA are not validated for the older population in Ethiopia. Due to lack of simple, and valid measurement approaches, older age populations are always excluded from research that investigates the burden of malnutrition in Ethiopia. For instance, the Demographic Health Survey of Ethiopia didn’t reveal older age malnutrition status, indicating they haven’t got due attention [12]. Identification of a more valid, reliable and practical tool to assess malnutrition status in older age people in a resource-limited settings is important so as to provide appropriate interventions [25].

This study was conducted with the aim of determining the reliability and validity of anthropometric measurements in identifying malnutrition status among older age people living in Borena District, South Wollo Zone, Ethiopia. This study will help health care providers to assess the malnutrition status of elders using appropriate tools and thus provide appropriate care to improve nutritional status.

## Methods

### Study design, area and participants

A community-based cross sectional study was conducted among older age people living in Borena District, South Wollo Zone, Ethiopia, between 25^th^ January and 5^th^ March 2020. Borena District is 180 km away from the zonal town, Dessie and 580 km away from Addis Ababa, the capital city of Ethiopia. The District administration has 39 Kebeles, (5 urban and 34 rural)s and the population in 2019/20 was estimated to be 186,173. Of these, 3.2%, that is 5958 people, were estimated to be aged 65 years and over [5].

All people aged 65 and over years old living in Borena District were potential study participants. The final subjects need to have been living in the Kebele that was randomly selected for at least 6 months. Those who were **e**dematous and/or severely ill were not included in this study.

The sample size was determined by using the prevalence of malnutrition among older age individuals in Gondar, Ethiopia which was previously determined by Mesele [5] to be 21.9%. The confidence interval was considered to be 5% of confidence level with a 1.5 design effect, 5% margin of error and 10% non-response. Hence, a total of 436 study subjects were included. A multi-stage sampling technique was then used to identify the study participants. A simple random sampling technique was used to select 12 Kebeles from 39 District Kebeles. Households with which had people aged 65 years and above were identified from Health Extension Worker’s family folder in the respective Kebeles. The sample was then proportionally allocated, depending on the number of people aged 65 years and over in each of the randomly selected 12 Kebeles. Finally, a systematic random sampling technique, which include every 12 households, was used to select study subjects. When there were more than one elder in one house, one elder was elected by lottery method.

### Data collections and measurements

A pre-tested structured interviewer administered questionnaire that comprised of socioeconomic, demographic, environmental, and diet-related measures was used. Anthropometric measures were also taken. Five data collectors were trained to collect the data.

Malnutrition was assessed using the full MNA tool which comprised of anthropometric measurements, recent dietary intake, general condition and subjective assessments [13, 25]. A total of 18 items were used to generate a score with a minimum of zero and a maximum of 30 points. Based on the score, the malnutrition status was classified as malnourished, at risk of malnutrition and well-nourished when the score was less than 17, 17–23.5, and greater than 23.5, respectively [13, 26].

### Anthropometric measurements

Height was measured using portable height stadiometer. Participants were barefoot, legs straight, shoulders relaxed and look straight ahead in the horizontal plane. Participants were also asked to inhale deeply, hold their breath and maintain an erect position prior to taking the measurement. Height measurement was taken to the nearest 0.1 cm. For those people with a kyphosis, thus making it difficult to measure height, the arm span was used as an alternative.The arm span is the distance from the middle finger tip of the left hand to that of right hand when both arms are stretched out horizontally. This length is then converted to a height equivalent [33]. Weight was measured with a portable digital weight scale. Participants wore minimum clothing and stood in the middle of the scale’s platform. Reading of weight was approximated to the nearest 0.1 kg. All measurements were taken three times and then averaged. Body Mass Index (BMI) is calculated as body weight in kilograms divided by height in meters squarred. In this study, nutritional status was considered as normal when the BMI was between 18.5 kg/m2 and 24.9 kg/m2 and malnourished when BMI was less than 18.5 kg/m2 [23].

To measure participants’ MUAC, non-stretch adult MUAC tape was used. The left arm was flexed at the elbow to 90 degree, and the midpoint between the lateral acromion and distal olecranon identified and marked. Then the arm was relaxed and the MUAC tape placed around the marked midpoint of the arm. The measurement was recorded to the nearest 0.1 cm. An MUAC of less than 21 cm was considered as malnourished [34].

The CC was measured at the widest point of the calf circumference, between the ankle and knee in a sitting position with the leg bent 90° at the knee to the nearest 0.1 cm using non-stretchable tape. The tape was manipulated to maintain close contact with the skin without compressiing the underlying tissues. A CC less than 31 cm was considered to be malnourished [27].

### Data quality management

A questionnaire was prepared in English and translated into Amharic for the field work and then back to English for checking the language consistency. The interviewers and supervisors were trained in data collection, interviewing and measurement techniques for two days. A pre-test (from 5% of older age people from non-seleced Kebeles) was undertaken prior to undertaking the data collection work to assess the consistence of responses, technical measurement error, precision and accuracy of anthropometric measurements. A digital scale was used to reduce frequent calibration and was standardized with a constant weight. The collected information was reviewed on a daily basis and possible errors were returned to the data collectors for correction.

### Statistical analysis

The data were entered into Epi-Data version 3.1, and after cleaning and coding, imported to STATA version 15 (STATA Corp, College Station, Tx) for analysis. MNA was computed from the 18- items and used as a reference to compare the reliability and validity of BMI, MUAC and CC. The data wre explored for missing values and outliers before categoring variables. Characteristics of study subjects were expressed using frequency and percentage; and differences in distribution were demonstrated for categorical variables using Pearson’s chi-square test.

The reliability of the BMI, MUAC and CC was calculated using coefficient of Cronbach’s α and a Cronbach’s α value of 0.60, 0.70 & 0.80 were considered acceptable, adequate and good respectively [21]. Criterion related validity of the tools were checked after identifying a significant positive Pearson correlation between total MNA score and the single anthropometric measurement of BMI, MUAC, and CC. Compared to the MNA, the overall accuracy of BMI, MUAC, and CC were assessed using the Area under Receiver Operating Characteristic (ROC) curve (AUC). If the area under curve (AUC) was 0.50, no acceptable combinations of sensitivity and specificity could be found. As a general rule of thumb, an AUC of 0.60 was considered not acceptable; above 0.70, good; higher than 0.80, very good, and greater than 0.90 excellent [21].

Sensitivity and specificity were calculated using (ROC) curves. The Youden Index (Sensitivity + specificity—1) was used to conclude the best cut-off point of BMI, MUAC and CC [12].

### Ethical considerations

Ethical approval was obtained from the Research Ethics Review Committee of the College of Health Sciences and Medicine, Wollo University with reference number WU-CMHS-3412/08/2019. All methods were carried out in accordance with the Declaration of Helsinki and regulations. Informed written consent to participate was obtained from each selected participant confirmed by their signinature for those who are literate and by their finger stamp for illiterate participants. Participants were advised that they were free to withdraw or discontinue participation at any time without any form of prejudice. Privacy and confidentiality of the collected information was maintaned throughout the process. All information gained during the study was kept strictly confidential.

## Results

### Sociodemographic characteristics of participants

The overall response rate for the study was 96.6%. The characteristics of the sample are presented in Table 1. The mean age of participants was 68.2 (SD 2.8) years and the majority (78.9%) were aged between 65–69 years of age. Overall, 49.6% of participants were male, and more than half of the participants (53%) were Orthodox Christians, and the rest Muslims. Overall, 78.1% of the study subjects were unable to read and write and nearly two-thirds (67%) were married (Table 1). More men were able to read and write than women (27.8% vs 9%, *p*-value < 0.001), while more women has no formal education (88.7% vs 67.5%) and higher proportion of women were widowed than men (31.1% vs 17.7%, *p*-value = 0.005). The proportion of partners who were able to read and write are higher among women than men (14.6% vs 6%, *p*-value = 0.011).

**Table 1:** Sociodemographic characteristics of older age people in Borena District, South Wollo Zone, Ethiopia in March 2020

Variables	Men(*n* = 209) Frequency (%)	Women(*n* = 212) Frequency (%)	Total(*n* = 421) Frequency (%)	*P*-value
**Age group in years**				
65–69 years	161(77)	171(80.7)	332(78.9)	0.310
70–74 years	37(17.7)	35(16.5)	72(17.1)	
75–80 years	11(5.3)	6(2.9)	17(4)	
**Religion**				
Orthodox	111(53.1)	112(52.8)	223(53)	0.954
Muslim	98(46.9)	100(47.2)	198(47)	
**Education Status of Respondent**				
No formal education	141(67.5)	188(88.7)	329(78.1)	0.0001
Read and write	58(27.8)	19(9)	77(18.3)	
Primary and Secondary education	10(3.8)	5(2.4)	15(3.1)	
**Marital status**				
Married	151(72.3)	131(61.8)	282(67)	0.005
Widowed	37(17.7)	66(31.1)	103(24.5)	
Single/divorced/separated	21(10.0)	15(7.1)	36(8.5)	
**Educational status of partner (*n* = 281)**				
No formal Education	142(94.0)	111(85.4)	253(90)	0.011
Read and write	9(6)	19(14.6)	28(10.0)	
**Monthly income**				
< 100 birr	23(11.0)	34(16.0)	57(13.5)	0.509
100–500 birr	158(75.6)	159(75.0)	317(75.3)	
501–1000 birr	28(13.4)	19(9.0)	47(11.2)	

### Nutritional findings based on BMI, MUAC, CC, and MNA assessments

Based on an MNA score of < 17, 71.5% (95% CI 67.2–75.3) of respondents were malnourished and 16.2% (95% CI 11.4–19.7) were at risk of malnutrition. Based on a MUAC of < 21 cm, 58.7% were malnourished, whereas 65.8% were malnourished based on CC < 31 cm. Based on BMI of < 18.5 kg/m^2^, 69.1% were malnourished (Table 2). In addition, females were relatively more malnourished than males (74.5% compared to 68.4%, *p*-value < 0.009) based on the MNA score but no association was observed between sex and malnutrition status by BMI, MUAC and CC.

**Table 2:** Malnutrition status of older age individuals based on BMI, MUAC, CC and MNA score in Borena District, South Wollo Zone, Ethiopia March 2020 (*n* = 421)

Variable	Nutritional Status	Men n (%)	Women n (%)	*P*-value
**MUAC**	Normal(> = 21 cm)	86(41.2)	92(43.4)	0.64
	malnourished (< 21 cm)	123(58.8)	120(56.6)	
**CC**	Normal(> = 31 cm)	69(33.1)	64(30.2)	0.53
	malnourished (< 31 cm)	140(66.9)	148(69.8)	
**BMI**	Normal (> = 18.5)	76(36.4)	55(26.0)	0.074
	CED (< 18.5 kg/m. sq)	133(63.6)	157(74.0)	
**MNA**	Normal (> = 24)	36(17.3)	16(7.5)	0.009
	At risk of malnutrition (17–23.5)	30(14.3)	38(17.9)	
	malnourished (< 17)	143(68.4)	158(74.5)	

*CED* Chronic energy deficiency, *MNA* Mini Nutritional Assessment, *BMI* Body Mass Index, *MUAC* Mid Upper Arm Circumference, *CC* Calf Circumference, *r* Pearson correlation

### Reliability and validity of anthropometric measurements (BMI, MUAC, and CC)

**Fig. 1:**
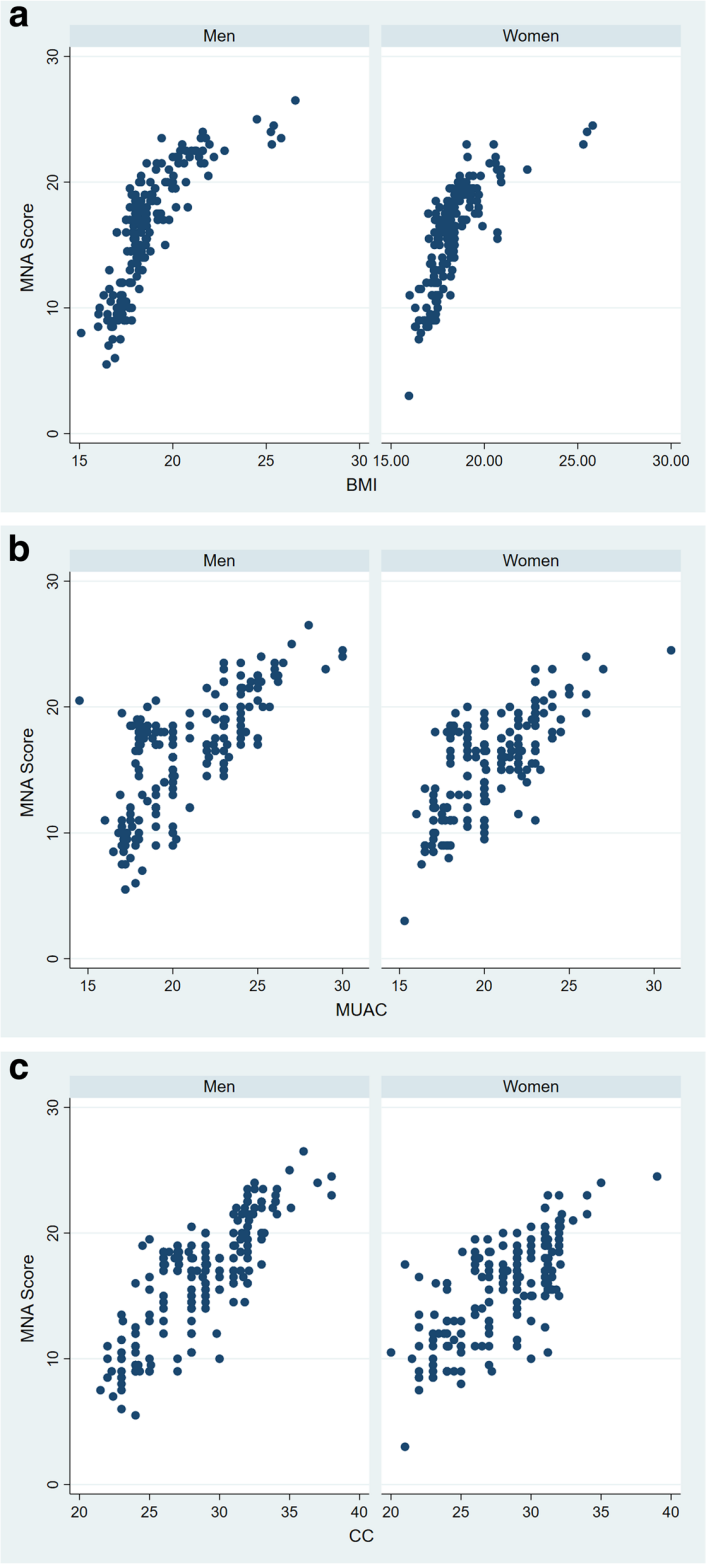
**a**-**c**: The correlation of MNA and anthropometric measurements a) MNA with BMI, **b**) MNA with MUAC and **c**) MNA with CC) among older people in Borena District, South Wollo Zone, Ethiopia March 2020(*n* = 421)

The internal consistency of the three anthropometric measurements as measured by Cronbach’s α coefficient was found to be 0.847**.** However, deleting one of the BMI, MUAC or CC from the measurement significantly lowers the Cronbach’s α value to 0.783, 0.777 and 0.792 respectively. Anthropometric measurements (BMI, MUAC, and CC) and the total MNA were correlated to show the criterion related validity of the tool (between MNA, BMI(*r* = 0.562, *p* < 0.01) (Fig. 1 a); MNA, MUAC(*r* = 0.433, *p* < 0.01(Fig. 1 b); and MNA, CC(*r* = 0.521, *p* < 0.01)( Fig. 1 c).

### Sensitivity and specificity of BMI, MUAC and CC

**Fig. 2:**
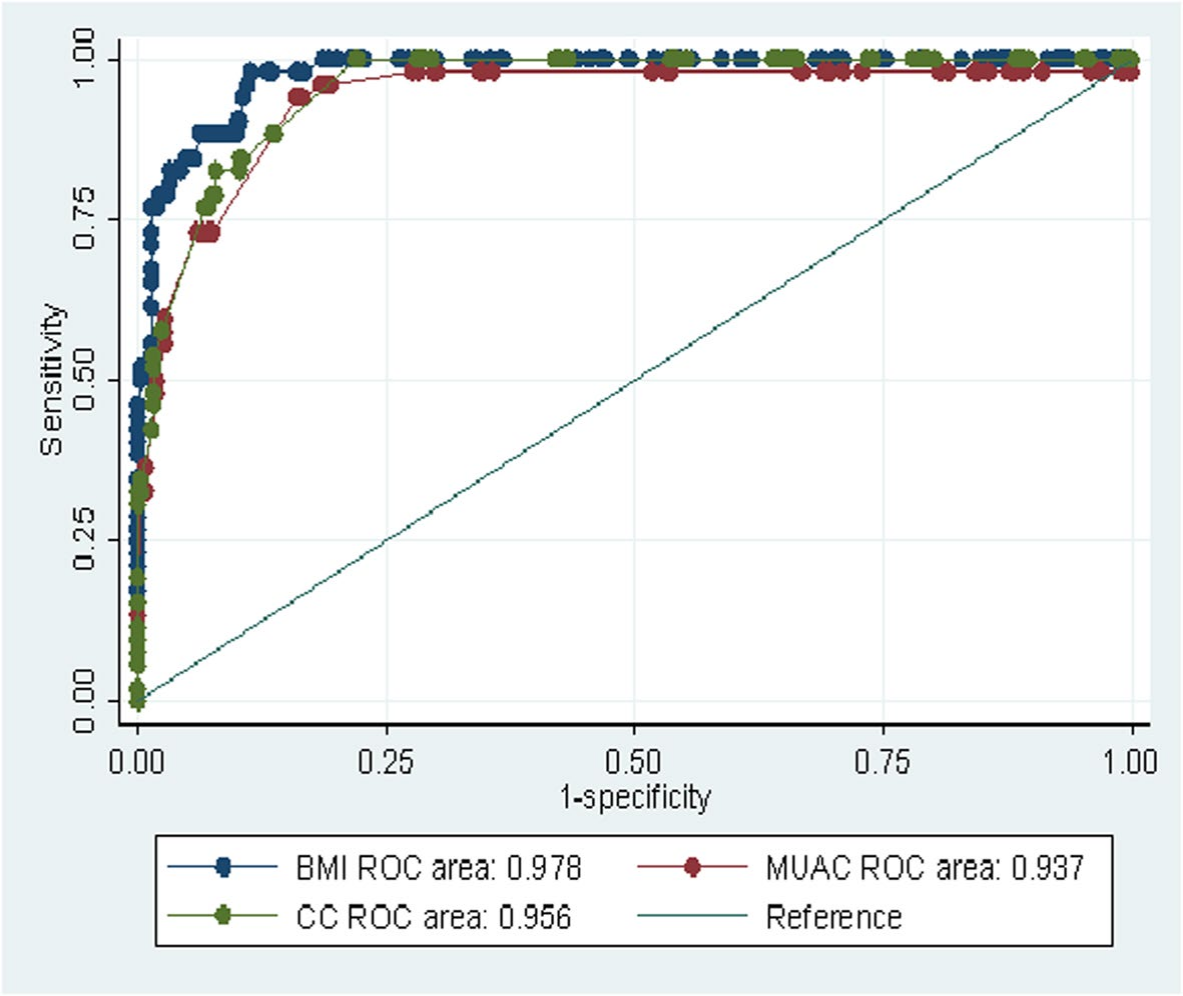
ROC curve analysis of BMI, MUAC and CC in predicting malnutrition among older people using MNA as reference in Borena District, South Wollo Zone, Ethiopia March 2020(*n* = 421)

**Fig. 3:**
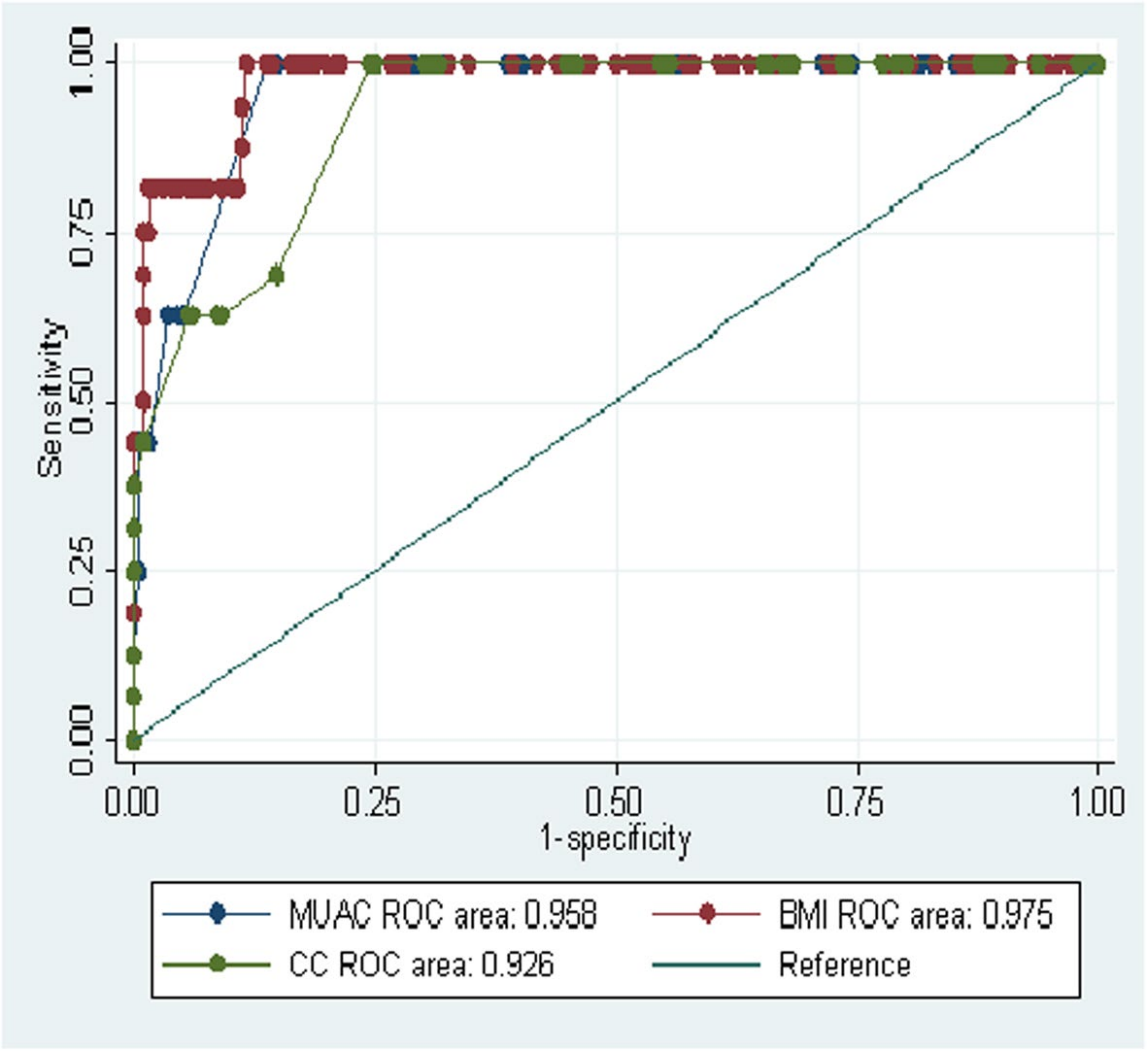
ROC curve analysis of BMI, MUAC and CC in predicting malnutrition among older women using MNA as reference in Borena District, South Wollo Zone, Ethiopia March 2020(*n* = 212)

**Fig. 4:**
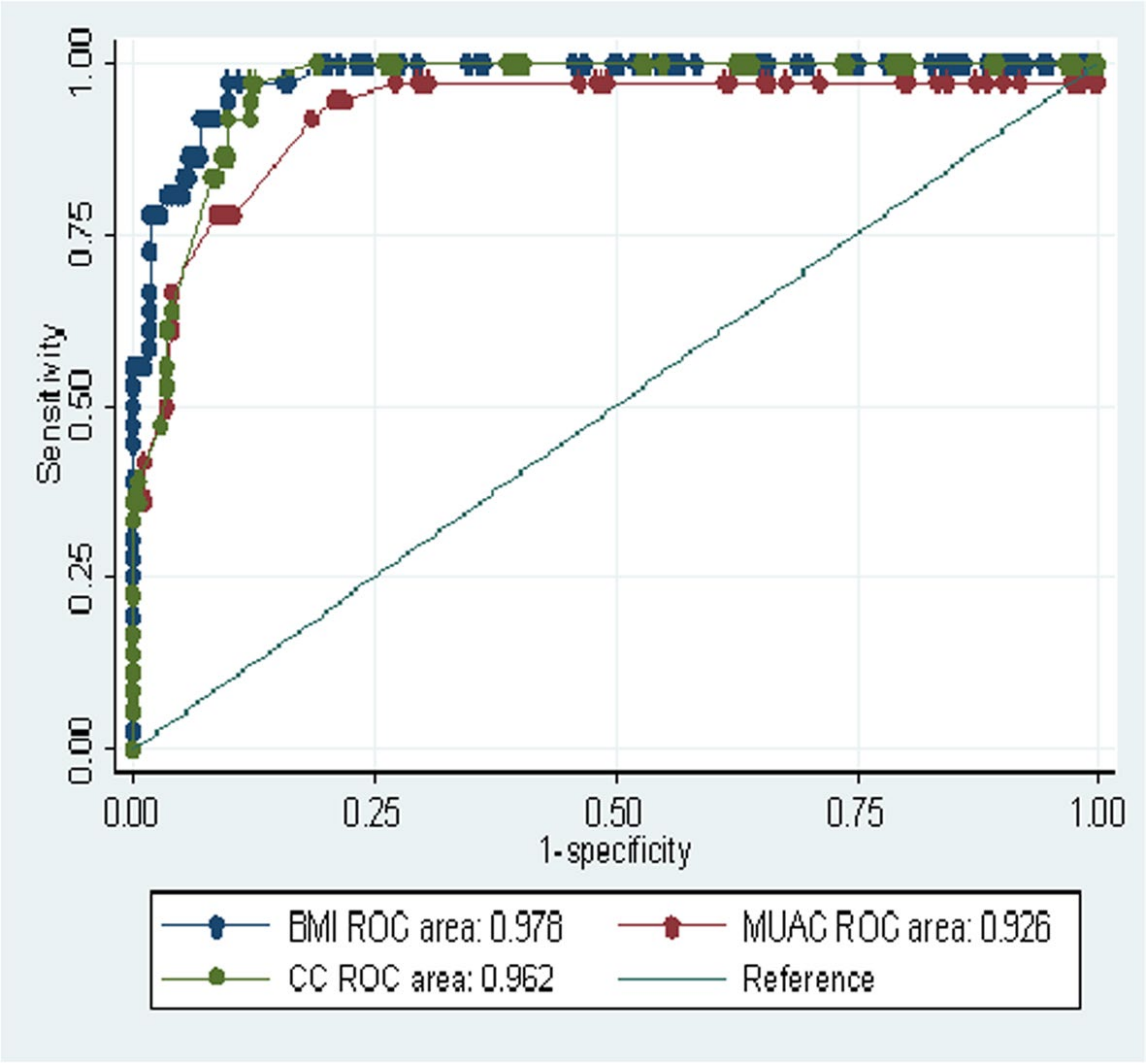
ROC curve analysis of BMI, MUAC and CC in predicting malnutrition among older men using MNA as reference in Borena District, South Wollo Zone, Ethiopia March 2020(*n* = 209)

The area under the curve (AUC) for each anthropometric measurement BMI, MUAC & CC were illustrated in Fig. 2 with the the overall accuracy of each measurement 0 0.978 (95% CI, 0.965–0.991, *p* < 0.001)), 0.937 (95% CI, 0.897–0.978, *p* < 0.001) and 0.956 (95% CI, 0.936–0.976, *p* < 0.001) in their place of order.**.** The overall accuracy of MUAC and CC showed a slight variation in women and men. MUAC has higher accuracy in screening malnutrition in women than men (Fig. 3) but CC has better accuracy in men than women ( Fig. 4).

Based on the established cut-off points using the ROC curve, the sensitivity and specificity of BMI, MUAC and CC in both sexes were found to be 90% ( 95% CI, 81.5%-98.5%), 96% (95% CI, 92.9%-100%); 78% (95% CI, 70.8%-85.1%) 94% (95% CI, 89.2%-99.7%) and 84% 95% CI, 76.2%-91–8%), 95% (95% CI, 90.4%-99.6%) respectively. Using the Youden index the best cut of points were 19 cm for MUAC and 30 cm for CC with their sensitivity and specificity of 88%(95% CI, 79.7%-96.3%), 86% (95% CI, 78%-94%), 92% (95% CI, 84.3%-100%) and 89% (95% CI, 80.6%-97.4%) in both sexes respectively. Using a MUAC of 19 cm for screening malnutrition among older age s increases the sensitivity from 78 to 88% (Suplementary Table 1 a). Similarly, a CC of 30 cm also increases the sensitivity from 84 to 92% (Suplementary Table 1 b). However, in case of BMI, the Youden index and the established cut-off point were the same (Suplementary Table 1 C). The optimal cut-off points were relatively the same for both men and women (Supplementary Table 2 a-d).

## Discussion

The current study determined the relaibility and accuracy of BMI, MUAC and CC against MNA screening tool among older age population in Ethiopia. The reliability of BMI, MUAC and CC using Cronbach’s alpha was found 0.847 which is acceptable and have very good internal consistency to assess the malnutrition status of elders [12, 23]. Significant positive correlations were observed between the full MNA and BMI, MUAC and CC. Previous studies showed similar findings in Thailand and Turkey [29, 30].

The overall accuracies of BMI, MUAC and CC using ROC curve were found to be in the excellent category in both sexes [11, 12, 23, 34]. Based on the established cut points, the sensitivity and specificity of BMI, MUAC and CC were classifed as very good [31]. This finding is similar to a study done in South India in 2014 [26, 31]. However, in the current study, reducing the cut-off points to 19 cm for MUAC and 30 cm for CC had resulted in an increase in sensitivity by 10% and 8% for MUAC and CC respectively and only a slight decrease in specificity. This adjustment of the cut-off point will help in efficient utilization of resources and older people will have the oppportunity to be included in nutrition intervention programs. While a geriatric assessment, using the MNA, is a more comprehensive approach, anthropometric measurements such as BMI, MUA and CC are easy to apply at a health facility or community level by health care provider when limited time exists. Older people, with malnutrition or at risk of malnutrition need comprehensive multidisciplinary nutrition intervention to support adequate dietary intake, maintain or increase body weight and/or improve functional and clinical outcomes [31, 35]. Studies from rural India, China, and Netherlands showed that MUAC and CC can be used as viable measure of malnutrition screening and diagnosis alternative to BMI, full-MNA and MNA-short form in older individuals [36–38]. This study also confirm that MUAC and CC have a very good and excellent internal consistency and accuracy in screening malnutrition among older individuals. Rourtine application of these tools will significantly reduce mortality associated with malnutrition and improve clinical prognosis of older population [39].

One of the strengths of this study is the validation of anthropometric measurements against the MNA score, which is a screening assessment tool, used both in community-dwelling [40] and geriatric settings [41, 42]. Moreover, MNA is a valid tool used to evaluate nutrient-rich food intake habits, functional status and cognitive function. A further strength of the study is conducted in rural community where the nutrition care practice and regular health screening services are neglected for older population and the validated tools are not complex and time consuming.

However, the current study has limitations in addressing biological biomarkers of malnutrition, examining usual nutrient intakes and concentration of albumin and other nutrients that may demonstrate the actual malnutrition situation in older population. Recall bias may be another source of error in the older age population that could lead to over or underestimation of malnutrition. The current study didn’t consider the effect cross-cultural adaptation of MNA tool in validating anthropometric measurements.

## Conclusion

The current study demonstrated that BMI has good sensitivity and specificity to identify malnutrition status among older people with the established cut-off points (BMI < 18.5 kg/m^2^), whereas and MUAC < 21 cm and CC of < 31 cm had a lower sensitivity, compared to the proposed these new cut-off points (MUAC < 19 cm and CC < 30 cm). In areas where the use of the MNA is impossible, application of BMI, MUAC or CC can address the health demands posed by older people with malnutrition, particularly in resource-limited countries where the cases to service providers ratio is high. This is likely to have large public health and clinical practice implications, especially with respect to nutritional interventions.

## Supplementary Information

**Additional file 1.**

## Data Availability

All data generated or analysed during this study are included in this published article [and its supplementary information files].

## Abbreviations

AUC: Area Under the Curve
BMI: Body Mass Index
CC: Calf Circumference
MNA: Mini-Nutritional Assessment;
MUAC: Mid-Upper Arm Circumference;
ROC: Receiver Operating Characteristic

## References

[1] World Health Organisation. 10 facts on ageing and the life course. 2012. http://www.who.int/features/factfiles/ageing/ageing_facts/en/index.html.

[2] Power L, Mullally D, Gibney ER, Clarke M, Visser M, Volkert D (2018). A review of the validity of malnutrition screening tools used in older adults in community and healthcare settings – a MaNuEL study. Clin Nutr ESPEN [Internet].

[3] United Nations Department of Economic and Social Affairs, Population Division. World Population Ageing 2020 Highlights: Living arrangements of older persons (ST/ESA/SER.A/451), 2020.

[4] United Nations Population Division. World Population Prospects 2019.

[5] Tessfamichael D, Gete AA, Wassie MM. High prevalence of undernutrition among elderly people in Northwest Ethiopia: a cross sectional study. J Nutrition Health Food Sci. 2014;2(4):1–5. 10.15226/jnhfs.2014.00131.

[6] Leslie W, Hankey C (2015). Aging, nutritional status and health. Healthcare.

[7] Help Age International (2013). Vulnerability of Older People in Ethiopia: The Case of Oromia, Amhara and SNNP Regional States.

[8] Pearce, S. Nutrition and Malnutrition in the Older age . 2014. Available from: http://www.uth.tmc.edu/hgec/GemsAndPearls/healthPromotions_Nutrition.html. [cited December 7, 2018.

[9] Cawood A, Elia M, Stratton R (2012). Systematic review and meta-analysis of the effects of high protein oral nutritional supplements. Ageing Res Rev.

[10] Söderström L (2013). Nutritional status among older people Risk factors and consequences of malnutrition, in Department of public health and caring sciences, clinical nutrition and metabolism.

[11] Ghimire S, Baral BK, Callahan K. Nutritional assessment of community-dwelling older adults in rural Nepal. PLOS ONE. 2017;12(2):e0172052. 10.1371/journal.pone.0172052. 10.1371/journal.pone.0172052 PMC5308814 28196115

[12] Hailemariam H, Singh P, Fekadu T (2016). Evaluation of mini nutrition assessment (MNA) tool among community dwelling older age in urban community of Hawassa city. Southern Ethiopia. BMC Nutr..

[13] Guigoz Y, Vellas B, Garry PJ. Mini nutritional assessment: a practical assessment tool for grading the nutritional state of elderly patients. In: Vellas B, editor. The Mini Nutritional Assessment (MNA), Supplement No 2. Paris: Serdi Publisher; 1994. p. 15–59.

[14] Joaquin C, Puig R, Gastelurrutia P, Lupón J, de Antonio M (2019). Mini nutritional assessment is a better predictor of mortality than subjective global assessment in heart failure out-patients. Clin Nutr.

[15] Saghafi-Asl M, Vaghef-Mehrabany E, Karamzad N, Daeiefarshbaf L, Kalejahi P, Asghari-Jafarabadi M (2018). Geriatric nutritional risk index as a simple tool for assessment of malnutrition among geriatrics in Northwest of Iran: comparison with mini nutritional assessment. Aging Clin Exp Res.

[16] Ye X-J, Ji Y-B, Ma B-W, Huang D-D, Chen W-Z (2018). Comparison of three common nutritional screening tools with the new European Society for Clinical Nutrition and Metabolism (ESPEN) criteria for malnutrition among patients with geriatric gastrointestinal cancer: a prospective study in China. BMJ Open.

[17] Marshall S, Craven D, Kelly J, Isenring E (2018). A systematic review and meta-analysis of the criterion validity of nutrition assessment tools for diagnosing protein-energy malnutrition in the older community setting (the MACRo study). Clin Nutr.

[18] Cascio BL, Logomarsino JV (2018). Evaluating the effectiveness of five screening tools used to identify malnutrition risk in hospitalized elderly: A systematic review. Geriatr Nurs.

[19] Becker L, Volkert D, Christian Sieber C, Gaßmann K-G, Ritt M (2019). Predictability of a modified Mini- Nutritional- Assessment version on six-month and one-year mortality in hospitalized geriatric patients: a comparative analysis. Sci Rep.

[20] Thomas J, Kaambwa B, Delaney C, Miller M (2019). 2An evaluation of the validity of nutrition screening and assessment tools in patients admitted to a vascular surgery unit. Br J Nutr.

[21] Diekmann R, Winning K, Uter W, Kaiser MJ, Sieber CC (2013). Screening for malnutrition among nursing home residents — a comparative analysis of the mini nutritional assessment, the nutritional risk screening, and the malnutrition universal screening tool. J Nutr Health Aging.

[22] Marshall S, Young A, Bauer J, Isenring E (2016). Malnutrition in geriatric rehabilitation: prevalence, patient outcomes, and criterion validity of the scored patient-generated subjective global assessment and the mini nutritional assessment. J Acad Nutr Diet.

[23] Mesfin Agachew Woldekidan (2021). Demewoz Haile, Bilal Shikur & Seifu Hagos Gebreyesus Validity of Mini Nutritional Assessment tool among an elderly population in Yeka sub-city, Addis Ababa, Ethiopia. , South Afr J Clin Nutr.

[24] Abrhame T, Haidar J (2015). The sensitivity and specificity of mid-upper arm circumference compared to body mass index in screening malnutrition of adult HIV patients taking art evidence from selected facilities of Addis Ababa, Ethiopia. Sci J Public Health.

[25] Wijnhoven HAH, Schilp J, van Bokhorst-de van der Schueren MAE, de Vet HCW, Kruizenga HM, Deeg DJH, et al. Development and validation of criteria for determining undernutrition in community-dwelling older men and women: The Short Nutritional Assessment Questionnaire 65 +. Clin Nutr. 2012;31(3):351–8. 10.1016/j.clnu.2011.10.013. 10.1016/j.clnu.2011.10.013 PMC6121713 22119209

[26] Suzana S, Siti Saifa H (2007). Validation of nutritional screening tools against anthropometric and functional assessments among elderly people in Selangor. Malays J Nutr..

[27] Miranda D, Cardoso R, Gomes R, Guimarães I, de Abreu D, Godinho C, Pereira P, Domingos J, Pona N, Ferreira JJ (2016). Undernutrition in institutionalized elderly patients with neurological diseases: comparison between different diagnostic criteria. J Nurs Home Res Sci (JNHRS).

[28] Soini H, Routasalo P, Lagstrom H (2004). Characteristics of the mini-nutritional assessment in older age home-care patients. Eur J Clin Nutr.

[29] Sukkriang Naparat, Somrak Kamlai (2021). Correlation between mini nutritional assessment and anthropometric measurements among community-dwelling older age individuals in rural Southern Thailand. J Multidiscip Healthc.

[30] Başıbüyük GO, Ayremlou P, Saeidlou SN, Ay F, Dalkıran A (2021). A comparison of the different anthropometric indices for assessing malnutrition among older people in Turkey: a large population-based screening. J Health Popul Nutr.

[31] Jose S, Kumari KS, Jose S (2020). Validity assessment of MNA among an older age population in Kerala. South India.

[32] Guigoz Y, Vellas B (2021). Nutritional assessment in older adults: MNA® 25 years of a screening tool & a reference standard for care and research; what next?. J Nutr Health Aging.

[33] Rabito EI, Mialich MS, Martınez EZ, Garcıa RWD, Jordao AA, Marchini JS (2008). Validation of predictive equations for weight and height using a metric tape. Nutr Hosp..

[34] Government of Ethiopia, Federal Ministry of Health. National guideline for the management of acute malnutrition. Addis Ababa: FMOH; 2019. http://repository.iifphc.org/handle/123456789/1023.

[35] Volkert D, Beck MA, Cederholm T, Cereda E, Cruz-Jentoft A (2019). Management of malnutrition in older patients—current approaches, evidence and open questions. J Clin Med.

[36] Garg S, Biswas B, Maharana SP, Dasgupta A (2018). Potential of mid-upper arm circumference to replace body mass index as a screening tool for assessment of nutritional status: a study among a rural elderly population in eastern India. Natl J Physiol Pharm Pharmacol.

[37] Weng C-H, Tien C-P, Li C-I (2018). Mid-upper arm circumference, calf circumference and mortality in Chinese long-term care facility residents: a prospective cohort study. BMJ Open.

[38] Schaap LA (2018). Changes in body mass index and mid-upper arm circumference in relation to all-cause mortality in older adults. Clin Nutr..

[39] van Doorn-van Atten MN, de Groot LC, Romea AC, Schwartz S, de Vries JH, Haveman-Nies A (2019). Implementation of a multicomponent telemonitoring intervention to improve nutritional status of community-dwelling older adults: a process evaluation. Public Health Nutr.

[40] Phillips MB, Foley AL, Barnard R, Isenring EA, Miller MD (2010). Nutritional screening in community-dwelling older adults: a systematic literature review. Asia Pac J Clin Nutr.

[41] Sanchez-Rodriguez D, Annweiler C, Marco E, Hope S, Piotrowicz K (2020). European academy for medicine of ageing session participants’ report on malnutrition assessment and diagnostic methods; an international survey. Clin Nutr ESPEN.

[42] Camina-Martín MA, de Mateo-Silleras B, Malafarina V, Lopez-Mongil R, Niño- Martín V (2015). Nutritional status assessment in geriatrics: Consensus declaration by the Spanish society of geriatrics and gerontology nutrition work group. Maturitas.

