# Supplementary material for: Comparing the validity of anthropometric measurements in identifying malnutrition status of older age people in Borena district, North Central Ethiopia: a cross_sectional study
Source: BMC Geriatr. 2022 Oct 3;22:776. doi: 10.1186/s12877-022-03467-9 (PMC9528122; doi:10.1186/s12877-022-03467-9)
Supplement: Supplementary file 1 — Additional file 1. [file 12877_2022_3467_MOESM1_ESM.docx]

Supplementary Table 1 a: A ROC analysis for determining the best optimal cut-off points MUAC in reference to MNA to screen malnutrition among older adults in Borena District, South Wollo Zone, Ethiopia, March 2020 (n=421).

| MUAC | Sensitivity | 1 - Specificity | Specificity | Youden index |  |
| --- | --- | --- | --- | --- | --- |
| 19.32 | 0.884 | 0.139 | 0.865 | 0.75 |  |
| 22.9 | 0.84 | 0.128 | 0.872 | 0.72 |  |
| 22.45 | 0.816 | 0.114 | 0.886 | 0.702 |  |
| 22.65 | 0.823 | 0.088 | 0.912 | 0.735 |  |
| 22.3 | 0.796 | 0.048 | 0.952 | 0.745 |  |
| 22.16 | 0.808 | 0.058 | 0.942 | 0.750 |  |
| 22.06 | 0.805 | 0.078 | 0.922 | 0.727 |  |
| 21.8 | 0.724 | 0.019 | 0.981 | 0.705 |  |
| 21.55 | 0.718 | 0.019 | 0.981 | 0.699 |  |
| 21.35 | 0.702 | 0.019 | 0.981 | 0.683 |  |
| 21.1 | 0.699 | 0.019 | 0.981 | 0.68 |  |
| 23.75 | 0.94 | 0.269 | 0.731 | 0.671 |  |
| 23.4 | 0.935 | 0.269 | 0.731 | 0.666 |  |
| 23.25 | 0.93 | 0.269 | 0.731 | 0.661 |  |
| 23.15 | 0.927 | 0.269 | 0.731 | 0.658 |  |
| 23.05 | 0.924 | 0.269 | 0.731 | 0.655 |  |
| 20.6 | 0.656 | 0.019 | 0.981 | 0.637 |  |
| 20.15 | 0.653 | 0.019 | 0.981 | 0.634 |  |
| 20.05 | 0.642 | 0.019 | 0.981 | 0.623 |  |
| 24.05 | 0.973 | 0.404 | 0.596 | 0.569 |  |
| 24.2 | 0.973 | 0.423 | 0.577 | 0.55 |  |
| 24.4 | 0.973 | 0.442 | 0.558 | 0.531 |  |
| 24.55 | 0.981 | 0.5 | 0.5 | 0.481 |  |
| 19.75 | 0.482 | 0.019 | 0.981 | 0.463 |  |
| 24.8 | 0.981 | 0.519 | 0.481 | 0.462 |  |
| 19.4 | 0.469 | 0.019 | 0.981 | 0.45 |  |
| 19.25 | 0.466 | 0.019 | 0.981 | 0.447 |  |
| 19.1 | 0.463 | 0.019 | 0.981 | 0.444 |  |
| 25.1 | 0.992 | 0.635 | 0.365 | 0.357 |  |
| 25.85 | 0.997 | 0.673 | 0.327 | 0.324 |  |
| 25.5 | 0.995 | 0.673 | 0.327 | 0.322 |  |
| 25.25 | 0.992 | 0.673 | 0.327 | 0.319 |  |
| 18.9 | 0.333 | 0.019 | 0.981 | 0.314 |  |
| 18.65 | 0.331 | 0.019 | 0.981 | 0.312 |  |
| 18.4 | 0.309 | 0.019 | 0.981 | 0.29 |  |
| 18.25 | 0.304 | 0.019 | 0.981 | 0.285 |  |
| 18.15 | 0.29 | 0.019 | 0.981 | 0.271 |  |
| 18.05 | 0.271 | 0.019 | 0.981 | 0.252 |  |
| 26.1 | 1 | 0.808 | 0.192 | 0.192 |  |
| 17.95 | 0.192 | 0.019 | 0.981 | 0.173 |  |
| 17.85 | 0.184 | 0.019 | 0.981 | 0.165 |  |
| 26.35 | 1 | 0.846 | 0.154 | 0.154 |  |
| 17.75 | 0.157 | 0.019 | 0.981 | 0.138 |  |
| 17.65 | 0.154 | 0.019 | 0.981 | 0.135 |  |
| 26.75 | 1 | 0.865 | 0.135 | 0.135 |  |
| 17.55 | 0.144 | 0.019 | 0.981 | 0.125 |  |
| 17.4 | 0.122 | 0.019 | 0.981 | 0.103 |  |
| 17.25 | 0.117 | 0.019 | 0.981 | 0.098 |  |
| 27.5 | 1 | 0.904 | 0.096 | 0.096 |  |
| 17.15 | 0.108 | 0.019 | 0.981 | 0.089 |  |
| 28.5 | 1 | 0.923 | 0.077 | 0.077 |  |
| 17.05 | 0.089 | 0.019 | 0.981 | 0.07 |  |
| 29.5 | 1 | 0.942 | 0.058 | 0.058 |  |
| 16.95 | 0.041 | 0.019 | 0.981 | 0.022 |  |
| 30.5 | 1 | 0.981 | 0.019 | 0.019 |  |
| 16.85 | 0.033 | 0.019 | 0.981 | 0.014 |  |
| 16.65 | 0.03 | 0.019 | 0.981 | 0.011 |  |
| 13.5 | 0 | 0 | 1 | 0 |  |
| 32 | 1 | 1 | 0 | 0 |  |
| 16.4 | 0.011 | 0.019 | 0.981 | -0.008 |  |
| 16.15 | 0.008 | 0.019 | 0.981 | -0.011 |  |
| 15.65 | 0.003 | 0.019 | 0.981 | -0.016 |  |
| 14.9 | 0 | 0.019 | 0.981 | -0.019 |  |

Supplementary Table 1 b: A ROC analysis for determining the best optimal cut-off points CC in reference to MNA to screen malnutrition among older adults in Borena District, South Wollo Zone, Ethiopia, March 2020 (n=421).

| CC | Sensitivity | 1 - Specificity | Specificity | Youden index |
| --- | --- | --- | --- | --- |
| 30.05 | 0.916 | 0.113 | 0.887 | 0.803 |
| 25.05 | 0.445 | 0.014 | 0.986 | 0.431 |
| 25.55 | 0.45 | 0.019 | 0.981 | 0.431 |
| 26.05 | 0.493 | 0.099 | 0.901 | 0.394 |
| 28.05 | 0.694 | 0.302 | 0.698 | 0.392 |
| 26.15 | 0.493 | 0.104 | 0.896 | 0.389 |
| 26.65 | 0.507 | 0.118 | 0.882 | 0.389 |
| 28.2 | 0.694 | 0.307 | 0.693 | 0.387 |
| 26.25 | 0.493 | 0.108 | 0.892 | 0.385 |
| 26.85 | 0.507 | 0.123 | 0.877 | 0.384 |
| 26.35 | 0.493 | 0.113 | 0.887 | 0.38 |
| 26.95 | 0.507 | 0.127 | 0.873 | 0.38 |
| 28.9 | 0.699 | 0.321 | 0.679 | 0.378 |
| 28.35 | 0.694 | 0.316 | 0.684 | 0.378 |
| 26.45 | 0.493 | 0.118 | 0.882 | 0.375 |
| 29.9 | 0.818 | 0.443 | 0.557 | 0.375 |
| 28.6 | 0.694 | 0.321 | 0.679 | 0.373 |
| 29.05 | 0.804 | 0.434 | 0.566 | 0.37 |
| 29.65 | 0.813 | 0.443 | 0.557 | 0.37 |
| 24.75 | 0.378 | 0.009 | 0.991 | 0.369 |
| 29.35 | 0.809 | 0.443 | 0.557 | 0.366 |
| 29.15 | 0.804 | 0.443 | 0.557 | 0.361 |
| 30.55 | 0.866 | 0.505 | 0.495 | 0.361 |
| 24.4 | 0.364 | 0.005 | 0.995 | 0.359 |
| 24.25 | 0.359 | 0.005 | 0.995 | 0.354 |
| 24.15 | 0.354 | 0.005 | 0.995 | 0.349 |
| 27.05 | 0.565 | 0.222 | 0.778 | 0.343 |
| 24.05 | 0.344 | 0.005 | 0.995 | 0.339 |
| 27.5 | 0.569 | 0.241 | 0.759 | 0.328 |
| 27.15 | 0.565 | 0.241 | 0.759 | 0.324 |
| 27.9 | 0.569 | 0.245 | 0.755 | 0.324 |
| 31.05 | 0.919 | 0.623 | 0.377 | 0.296 |
| 31.22 | 0.952 | 0.656 | 0.344 | 0.296 |
| 31.15 | 0.919 | 0.627 | 0.373 | 0.292 |
| 31.27 | 0.952 | 0.66 | 0.34 | 0.292 |
| 31.4 | 0.952 | 0.665 | 0.335 | 0.287 |
| 31.55 | 0.967 | 0.693 | 0.307 | 0.274 |
| 31.85 | 0.981 | 0.708 | 0.292 | 0.273 |
| 31.95 | 0.981 | 0.712 | 0.288 | 0.269 |
| 31.75 | 0.971 | 0.703 | 0.297 | 0.268 |
| 31.65 | 0.967 | 0.703 | 0.297 | 0.264 |
| 23.9 | 0.201 | 0.005 | 0.995 | 0.196 |
| 23.6 | 0.196 | 0.005 | 0.995 | 0.191 |
| 23.3 | 0.191 | 0.005 | 0.995 | 0.186 |
| 32.05 | 1 | 0.816 | 0.184 | 0.184 |
| 23.15 | 0.187 | 0.005 | 0.995 | 0.182 |
| 23.05 | 0.177 | 0.005 | 0.995 | 0.172 |
| 32.15 | 1 | 0.84 | 0.16 | 0.16 |
| 32.25 | 1 | 0.844 | 0.156 | 0.156 |
| 32.35 | 1 | 0.854 | 0.146 | 0.146 |
| 32.45 | 1 | 0.858 | 0.142 | 0.142 |
| 32.75 | 1 | 0.873 | 0.127 | 0.127 |
| 33.05 | 1 | 0.91 | 0.09 | 0.09 |
| 33.15 | 1 | 0.915 | 0.085 | 0.085 |
| 33.5 | 1 | 0.92 | 0.08 | 0.08 |
| 22.7 | 0.081 | 0.005 | 0.995 | 0.076 |
| 33.9 | 1 | 0.925 | 0.075 | 0.075 |
| 22.35 | 0.077 | 0.005 | 0.995 | 0.072 |
| 22.15 | 0.072 | 0.005 | 0.995 | 0.067 |
| 34.05 | 1 | 0.953 | 0.047 | 0.047 |
| 34.55 | 1 | 0.962 | 0.038 | 0.038 |
| 35.05 | 1 | 0.972 | 0.028 | 0.028 |
| 35.55 | 1 | 0.976 | 0.024 | 0.024 |
| 36.5 | 1 | 0.981 | 0.019 | 0.019 |
| 21.75 | 0.019 | 0.005 | 0.995 | 0.014 |
| 37.5 | 1 | 0.986 | 0.014 | 0.014 |
| 20.5 | 0.005 | 0 | 1 | 0.005 |
| 21.25 | 0.01 | 0.005 | 0.995 | 0.005 |
| 38.5 | 1 | 0.995 | 0.005 | 0.005 |
| 19 | 0 | 0 | 1 | 0 |
| 40 | 1 | 1 | 0 | 0 |

Supplementary Table 1 c: A ROC analysis for determining the best optimal cut-off points BMI in reference to MNA to screen malnutrition among older adults in Borena District, South Wollo Zone, Ethiopia, March 2020 (n=421).

| BMI | Sensitivity | 1 - Specificity | Specificity | Youden Index |
| --- | --- | --- | --- | --- |
| 18.486 | 0.903 | 0.058 | 0.942 | 0.856 |
| 18.965 | 0.886 | 0.031 | 0.969 | 0.855 |
| 18.885 | 0.87 | 0.019 | 0.981 | 0.851 |
| 19.04 | 0.889 | 0.038 | 0.962 | 0.851 |
| 18.865 | 0.867 | 0.019 | 0.981 | 0.848 |
| 18.83 | 0.864 | 0.019 | 0.981 | 0.845 |
| 19.075 | 0.894 | 0.058 | 0.942 | 0.836 |
| 19.385 | 0.938 | 0.115 | 0.885 | 0.823 |
| 18.775 | 0.84 | 0.019 | 0.981 | 0.821 |
| 19.365 | 0.935 | 0.115 | 0.885 | 0.82 |
| 18.715 | 0.837 | 0.019 | 0.981 | 0.818 |
| 19.33 | 0.932 | 0.115 | 0.885 | 0.817 |
| 19.28 | 0.93 | 0.115 | 0.885 | 0.815 |
| 18.595 | 0.813 | 0 | 1 | 0.813 |
| 18.64 | 0.832 | 0.019 | 0.981 | 0.813 |
| 19.245 | 0.924 | 0.115 | 0.885 | 0.809 |
| 18.57 | 0.808 | 0 | 1 | 0.808 |
| 19.215 | 0.921 | 0.115 | 0.885 | 0.806 |
| 19.135 | 0.9 | 0.096 | 0.904 | 0.804 |
| 19.115 | 0.897 | 0.096 | 0.904 | 0.801 |
| 18.525 | 0.799 | 0 | 1 | 0.799 |
| 19.195 | 0.913 | 0.115 | 0.885 | 0.798 |
| 19.595 | 0.951 | 0.154 | 0.846 | 0.797 |
| 19.545 | 0.949 | 0.154 | 0.846 | 0.795 |
| 19.745 | 0.967 | 0.173 | 0.827 | 0.794 |
| 19.49 | 0.946 | 0.154 | 0.846 | 0.792 |
| 19.17 | 0.905 | 0.115 | 0.885 | 0.79 |
| 19.44 | 0.943 | 0.154 | 0.846 | 0.789 |
| 19.695 | 0.962 | 0.173 | 0.827 | 0.789 |
| 18.48 | 0.786 | 0 | 1 | 0.786 |
| 19.145 | 0.9 | 0.115 | 0.885 | 0.785 |
| 19.645 | 0.957 | 0.173 | 0.827 | 0.784 |
| 18.455 | 0.783 | 0 | 1 | 0.783 |
| 18.445 | 0.778 | 0 | 1 | 0.778 |
| 18.43 | 0.775 | 0 | 1 | 0.775 |
| 19.795 | 0.967 | 0.192 | 0.808 | 0.775 |
| 18.41 | 0.772 | 0 | 1 | 0.772 |
| 19.985 | 0.978 | 0.212 | 0.788 | 0.766 |
| 19.955 | 0.976 | 0.212 | 0.788 | 0.764 |
| 19.92 | 0.973 | 0.212 | 0.788 | 0.761 |
| 19.85 | 0.97 | 0.212 | 0.788 | 0.758 |
| 20.155 | 0.986 | 0.231 | 0.769 | 0.755 |
| 20.085 | 0.984 | 0.231 | 0.769 | 0.753 |
| 20.015 | 0.981 | 0.231 | 0.769 | 0.75 |
| 18.395 | 0.737 | 0 | 1 | 0.737 |
| 18.38 | 0.734 | 0 | 1 | 0.734 |
| 18.365 | 0.732 | 0 | 1 | 0.732 |
| 18.355 | 0.726 | 0 | 1 | 0.726 |
| 18.34 | 0.721 | 0 | 1 | 0.721 |
| 20.225 | 0.986 | 0.269 | 0.731 | 0.717 |
| 18.315 | 0.699 | 0 | 1 | 0.699 |
| 20.29 | 0.986 | 0.288 | 0.712 | 0.698 |
| 18.295 | 0.664 | 0 | 1 | 0.664 |
| 18.285 | 0.659 | 0 | 1 | 0.659 |
| 20.35 | 0.986 | 0.327 | 0.673 | 0.659 |
| 18.265 | 0.645 | 0 | 1 | 0.645 |
| 20.45 | 0.986 | 0.346 | 0.654 | 0.64 |
| 18.245 | 0.634 | 0 | 1 | 0.634 |
| 18.22 | 0.631 | 0 | 1 | 0.631 |
| 20.55 | 0.986 | 0.385 | 0.615 | 0.601 |
| 18.19 | 0.577 | 0 | 1 | 0.577 |
| 18.17 | 0.558 | 0 | 1 | 0.558 |
| 18.15 | 0.545 | 0 | 1 | 0.545 |
| 20.64 | 0.986 | 0.442 | 0.558 | 0.544 |
| 18.135 | 0.534 | 0 | 1 | 0.534 |
| 18.125 | 0.531 | 0 | 1 | 0.531 |
| 18.11 | 0.528 | 0 | 1 | 0.528 |
| 20.69 | 0.986 | 0.462 | 0.538 | 0.524 |
| 20.83 | 0.997 | 0.481 | 0.519 | 0.516 |
| 20.75 | 0.995 | 0.481 | 0.519 | 0.514 |
| 18.095 | 0.507 | 0 | 1 | 0.507 |
| 18.08 | 0.504 | 0 | 1 | 0.504 |
| 20.88 | 0.997 | 0.5 | 0.5 | 0.497 |
| 18.065 | 0.48 | 0 | 1 | 0.48 |
| 18.055 | 0.466 | 0 | 1 | 0.466 |
| 18.04 | 0.463 | 0 | 1 | 0.463 |
| 20.95 | 1 | 0.538 | 0.462 | 0.462 |
| 18.025 | 0.461 | 0 | 1 | 0.461 |
| 18.015 | 0.455 | 0 | 1 | 0.455 |
| 18.005 | 0.453 | 0 | 1 | 0.453 |
| 17.995 | 0.442 | 0 | 1 | 0.442 |
| 21.09 | 1 | 0.558 | 0.442 | 0.442 |
| 21.215 | 1 | 0.577 | 0.423 | 0.423 |
| 17.945 | 0.412 | 0 | 1 | 0.412 |
| 21.325 | 1 | 0.596 | 0.404 | 0.404 |
| 17.895 | 0.393 | 0 | 1 | 0.393 |
| 17.87 | 0.39 | 0 | 1 | 0.39 |
| 21.45 | 1 | 0.615 | 0.385 | 0.385 |
| 17.825 | 0.382 | 0 | 1 | 0.382 |
| 17.79 | 0.355 | 0 | 1 | 0.355 |
| 17.765 | 0.35 | 0 | 1 | 0.35 |
| 21.55 | 1 | 0.654 | 0.346 | 0.346 |
| 17.725 | 0.341 | 0 | 1 | 0.341 |
| 17.685 | 0.317 | 0 | 1 | 0.317 |
| 17.635 | 0.312 | 0 | 1 | 0.312 |
| 21.64 | 1 | 0.692 | 0.308 | 0.308 |
| 17.585 | 0.298 | 0 | 1 | 0.298 |
| 17.565 | 0.295 | 0 | 1 | 0.295 |
| 17.53 | 0.29 | 0 | 1 | 0.29 |
| 21.725 | 1 | 0.712 | 0.288 | 0.288 |
| 21.835 | 1 | 0.731 | 0.269 | 0.269 |
| 17.48 | 0.252 | 0 | 1 | 0.252 |
| 21.93 | 1 | 0.75 | 0.25 | 0.25 |
| 17.43 | 0.247 | 0 | 1 | 0.247 |
| 22.08 | 1 | 0.769 | 0.231 | 0.231 |
| 17.395 | 0.214 | 0 | 1 | 0.214 |
| 22.25 | 1 | 0.788 | 0.212 | 0.212 |
| 17.375 | 0.211 | 0 | 1 | 0.211 |
| 17.345 | 0.203 | 0 | 1 | 0.203 |
| 17.315 | 0.198 | 0 | 1 | 0.198 |
| 22.535 | 1 | 0.808 | 0.192 | 0.192 |
| 23.635 | 1 | 0.827 | 0.173 | 0.173 |
| 17.25 | 0.171 | 0 | 1 | 0.171 |
| 24.875 | 1 | 0.846 | 0.154 | 0.154 |
| 17.195 | 0.152 | 0 | 1 | 0.152 |
| 17.185 | 0.146 | 0 | 1 | 0.146 |
| 17.175 | 0.141 | 0 | 1 | 0.141 |
| 17.16 | 0.138 | 0 | 1 | 0.138 |
| 17.125 | 0.136 | 0 | 1 | 0.136 |
| 25.275 | 1 | 0.865 | 0.135 | 0.135 |
| 17.085 | 0.13 | 0 | 1 | 0.13 |
| 17.055 | 0.127 | 0 | 1 | 0.127 |
| 17.035 | 0.125 | 0 | 1 | 0.125 |
| 17.015 | 0.122 | 0 | 1 | 0.122 |
| 16.95 | 0.103 | 0 | 1 | 0.103 |
| 25.35 | 1 | 0.904 | 0.096 | 0.096 |
| 16.85 | 0.084 | 0 | 1 | 0.084 |
| 25.45 | 1 | 0.923 | 0.077 | 0.077 |
| 16.795 | 0.076 | 0 | 1 | 0.076 |
| 16.785 | 0.073 | 0 | 1 | 0.073 |
| 16.765 | 0.07 | 0 | 1 | 0.07 |
| 16.725 | 0.068 | 0 | 1 | 0.068 |
| 16.685 | 0.065 | 0 | 1 | 0.065 |
| 16.635 | 0.062 | 0 | 1 | 0.062 |
| 25.65 | 1 | 0.942 | 0.058 | 0.058 |
| 16.58 | 0.049 | 0 | 1 | 0.049 |
| 16.53 | 0.046 | 0 | 1 | 0.046 |
| 16.495 | 0.033 | 0 | 1 | 0.033 |
| 16.47 | 0.03 | 0 | 1 | 0.03 |
| 16.39 | 0.027 | 0 | 1 | 0.027 |
| 16.315 | 0.024 | 0 | 1 | 0.024 |
| 26.18 | 1 | 0.981 | 0.019 | 0.019 |
| 16.295 | 0.019 | 0 | 1 | 0.019 |
| 16.185 | 0.016 | 0 | 1 | 0.016 |
| 16.05 | 0.014 | 0 | 1 | 0.014 |
| 16.01 | 0.011 | 0 | 1 | 0.011 |
| 15.98 | 0.005 | 0 | 1 | 0.005 |
| 15.525 | 0.003 | 0 | 1 | 0.003 |
| 14.09 | 0 | 0 | 1 | 0 |
| 27.56 | 1 | 1 | 0 | 0 |

Supplementary Table 2 a: A ROC analysis for determining the best optimal cut-off points MUAC in reference to MNA to screen malnutrition among older men in Borena District, South Wollo Zone, Ethiopia, March 2020 (n=209).

| Cut point | Sensitivity | 1-Specificity | Specificity | Youden Index |
| --- | --- | --- | --- | --- |
| (>= 19.47) | 0.8758 | 0.1364 | 0.8636 | 0.7394 |
| (>= 22.5) | 0.9444 | 0.2081 | 0.7919 | 0.7363 |
| (>= 23) | 0.9167 | 0.1850 | 0.8150 | 0.7317 |
| (>= 22.4) | 0.9444 | 0.2139 | 0.7861 | 0.7305 |
| (>= 22.12) | 0.9444 | 0.2197 | 0.7803 | 0.7247 |
| (>= 22) | 0.9722 | 0.2717 | 0.7283 | 0.7005 |
| (>= 24) | 0.7778 | 0.0867 | 0.9133 | 0.6911 |
| (>= 23.3) | 0.7778 | 0.0925 | 0.9075 | 0.6853 |
| (>= 23.2) | 0.7778 | 0.0983 | 0.9017 | 0.6795 |
| (>= 21) | 0.9722 | 0.2948 | 0.7052 | 0.6774 |
| (>= 23.1) | 0.7778 | 0.1040 | 0.8960 | 0.6738 |
| (>= 20.2) | 0.9722 | 0.3006 | 0.6994 | 0.6716 |
| (>= 20.1) | 0.9722 | 0.3064 | 0.6936 | 0.6658 |
| (>= 24.1) | 0.6667 | 0.0405 | 0.9595 | 0.6262 |
| (>= 24.3) | 0.6389 | 0.0405 | 0.9595 | 0.5984 |
| (>= 24.5) | 0.6111 | 0.0405 | 0.9595 | 0.5706 |
| (>= 20) | 0.9722 | 0.4624 | 0.5376 | 0.5098 |
| (>= 24.6) | 0.5278 | 0.0347 | 0.9653 | 0.4931 |
| (>= 19.5) | 0.9722 | 0.4798 | 0.5202 | 0.4924 |
| (>= 19.3) | 0.9722 | 0.4855 | 0.5145 | 0.4867 |
| (>= 19.8) | 0.9722 | 0.4913 | 0.5087 | 0.4809 |
| (>= 25) | 0.5000 | 0.0347 | 0.9653 | 0.4653 |
| (>= 25.2) | 0.4167 | 0.0116 | 0.9884 | 0.4051 |
| (>= 26) | 0.3611 | 0.0000 | 1.0000 | 0.3611 |
| (>= 19.7) | 0.9722 | 0.6127 | 0.3873 | 0.3595 |
| (>= 25.7) | 0.3611 | 0.0058 | 0.9942 | 0.3553 |
| (>= 18.8) | 0.9722 | 0.6185 | 0.3815 | 0.3537 |
| (>= 25.3) | 0.3611 | 0.0116 | 0.9884 | 0.3495 |
| (>= 18.5) | 0.9722 | 0.6532 | 0.3468 | 0.3190 |
| (>= 18.3) | 0.9722 | 0.6590 | 0.3410 | 0.3132 |
| (>= 18.2) | 0.9722 | 0.6763 | 0.3237 | 0.2959 |
| (>= 18.1) | 0.9722 | 0.7110 | 0.2890 | 0.2612 |
| (>= 26.2) | 0.2222 | 0.0000 | 1.0000 | 0.2222 |
| (>= 18) | 0.9722 | 0.7977 | 0.2023 | 0.1745 |
| (>= 17.9) | 0.9722 | 0.8035 | 0.1965 | 0.1687 |
| (>= 26.5) | 0.1667 | 0.0000 | 1.0000 | 0.1667 |
| (>= 17.8) | 0.9722 | 0.8324 | 0.1676 | 0.1398 |
| (>= 27) | 0.1389 | 0.0000 | 1.0000 | 0.1389 |
| (>= 17.6) | 0.9722 | 0.8439 | 0.1561 | 0.1283 |
| (>= 28) | 0.1111 | 0.0000 | 1.0000 | 0.1111 |
| (>= 17.5) | 0.9722 | 0.8728 | 0.1272 | 0.0994 |
| (>= 17.3) | 0.9722 | 0.8844 | 0.1156 | 0.0878 |
| (>= 29) | 0.0833 | 0.0000 | 1.0000 | 0.0833 |
| (>= 17.2) | 0.9722 | 0.9017 | 0.0983 | 0.0705 |
| (>= 30) | 0.0556 | 0.0000 | 1.0000 | 0.0556 |
| (>= 17.1) | 0.9722 | 0.9191 | 0.0809 | 0.0531 |
| (>= 17) | 0.9722 | 0.9711 | 0.0289 | 0.0011 |
| (>= 14.5) | 1.0000 | 1.0000 | 0.0000 | 0.0000 |
| (>= 30) | 0.0000 | 0.0000 | 1.0000 | 0.0000 |
| (>= 16.9) | 0.9722 | 0.9769 | 0.0231 | -0.0047 |
| (>= 16.8) | 0.9722 | 0.9827 | 0.0173 | -0.0105 |
| (>= 16.5) | 0.9722 | 0.9942 | 0.0058 | -0.0220 |
| (>= 16) | 0.9722 | 1.0000 | 0.0000 | -0.0278 |

Supplementary Table 2 b: A ROC analysis for determining the best optimal cut-off points CC in reference to MNA to screen malnutrition among older men in Borena District, South Wollo Zone, Ethiopia, March 2020 (n=209).

| Cut point | Sensitivity | 1-Specificity | Specificity | Youden Index |
| --- | --- | --- | --- | --- |
| (>= 30.4) | 0.9275 | 0.1055 | 0.8945 | 0.8220 |
| (>= 31.3) | 0.9414 | 0.1214 | 0.8786 | 0.8200 |
| (>= 31.6) | 0.9167 | 0.0983 | 0.9017 | 0.8184 |
| (>= 31.1) | 0.9422 | 0.1272 | 0.8728 | 0.8150 |
| (>= 31) | 1.0000 | 0.1908 | 0.8092 | 0.8092 |
| (>= 31.5) | 0.9167 | 0.1214 | 0.8786 | 0.7953 |
| (>= 31.8) | 0.8611 | 0.0925 | 0.9075 | 0.7686 |
| (>= 31.7) | 0.8611 | 0.0983 | 0.9017 | 0.7628 |
| (>= 32) | 0.8333 | 0.0809 | 0.9191 | 0.7524 |
| (>= 31.9) | 0.8333 | 0.0867 | 0.9133 | 0.7466 |
| (>= 30.6) | 1.0000 | 0.2601 | 0.7399 | 0.7399 |
| (>= 29.8) | 1.0000 | 0.2659 | 0.7341 | 0.7341 |
| (>= 29.1) | 1.0000 | 0.2717 | 0.7283 | 0.7283 |
| (>= 29) | 1.0000 | 0.3873 | 0.6127 | 0.6127 |
| (>= 28.8) | 1.0000 | 0.3931 | 0.6069 | 0.6069 |
| (>= 28.4) | 1.0000 | 0.3988 | 0.6012 | 0.6012 |
| (>= 32.1) | 0.6389 | 0.0405 | 0.9595 | 0.5984 |
| (>= 28.1) | 1.0000 | 0.4046 | 0.5954 | 0.5954 |
| (>= 32.3) | 0.6111 | 0.0347 | 0.9653 | 0.5764 |
| (>= 32.4) | 0.5556 | 0.0347 | 0.9653 | 0.5209 |
| (>= 32.5) | 0.5278 | 0.0347 | 0.9653 | 0.4931 |
| (>= 28) | 1.0000 | 0.5260 | 0.4740 | 0.4740 |
| (>= 27.8) | 1.0000 | 0.5318 | 0.4682 | 0.4682 |
| (>= 27.1) | 1.0000 | 0.5491 | 0.4509 | 0.4509 |
| (>= 33) | 0.4722 | 0.0289 | 0.9711 | 0.4433 |
| (>= 33.1) | 0.3889 | 0.0058 | 0.9942 | 0.3831 |
| (>= 27) | 1.0000 | 0.6243 | 0.3757 | 0.3757 |
| (>= 26.8) | 1.0000 | 0.6301 | 0.3699 | 0.3699 |
| (>= 26.4) | 1.0000 | 0.6358 | 0.3642 | 0.3642 |
| (>= 33.8) | 0.3611 | 0.0000 | 1.0000 | 0.3611 |
| (>= 26.1) | 1.0000 | 0.6416 | 0.3584 | 0.3584 |
| (>= 33.2) | 0.3611 | 0.0058 | 0.9942 | 0.3553 |
| (>= 34) | 0.3333 | 0.0000 | 1.0000 | 0.3333 |
| (>= 26) | 1.0000 | 0.7341 | 0.2659 | 0.2659 |
| (>= 25.1) | 1.0000 | 0.7399 | 0.2601 | 0.2601 |
| (>= 34.1) | 0.2222 | 0.0000 | 1.0000 | 0.2222 |
| (>= 25) | 1.0000 | 0.7861 | 0.2139 | 0.2139 |
| (>= 24.5) | 1.0000 | 0.7919 | 0.2081 | 0.2081 |
| (>= 24.3) | 1.0000 | 0.7977 | 0.2023 | 0.2023 |
| (>= 24.2) | 1.0000 | 0.8035 | 0.1965 | 0.1965 |
| (>= 35) | 0.1667 | 0.0000 | 1.0000 | 0.1667 |
| (>= 35.1) | 0.1389 | 0.0000 | 1.0000 | 0.1389 |
| (>= 36) | 0.1111 | 0.0000 | 1.0000 | 0.1111 |
| (>= 24) | 1.0000 | 0.8902 | 0.1098 | 0.1098 |
| (>= 23.1) | 1.0000 | 0.8960 | 0.1040 | 0.1040 |
| (>= 37) | 0.0833 | 0.0000 | 1.0000 | 0.0833 |
| (>= 38) | 0.0556 | 0.0000 | 1.0000 | 0.0556 |
| (>= 23) | 1.0000 | 0.9653 | 0.0347 | 0.0347 |
| (>= 22.4) | 1.0000 | 0.9711 | 0.0289 | 0.0289 |
| (>= 22.3) | 1.0000 | 0.9769 | 0.0231 | 0.0231 |
| (>= 22) | 1.0000 | 0.9942 | 0.0058 | 0.0058 |
| (>= 21.5) | 1.0000 | 1.0000 | 0.0000 | 0.0000 |
| (>= 38) | 0.0000 | 0.0000 | 1.0000 | 0.0000 |

Supplementary Table 2 c: A ROC analysis for determining the best optimal cut-off points MUAC in reference to MNA to screen malnutrition among older women in Borena District, South Wollo Zone, Ethiopia, March 2020 (n=212).

| Cut point | Sensitivity | 1-specificity | Specificity | Youden Index |
| --- | --- | --- | --- | --- |
| (>= 19.3) | 0.8786 | 0.1378 | 0.8622 | 0.7408 |
| (>= 22.2) | 0.9145 | 0.1743 | 0.8257 | 0.7402 |
| (>= 22.8) | 0.9100 | 0.1713 | 0.8287 | 0.7387 |
| (>= 22.5) | 0.9231 | 0.1933 | 0.8067 | 0.7298 |
| (>= 22) | 1.0000 | 0.2806 | 0.7194 | 0.7194 |
| (>= 21.6) | 1.0000 | 0.2908 | 0.7092 | 0.7092 |
| (>= 21.5) | 1.0000 | 0.3214 | 0.6786 | 0.6786 |
| (>= 21.2) | 1.0000 | 0.3265 | 0.6735 | 0.6735 |
| (>= 21) | 1.0000 | 0.3878 | 0.6122 | 0.6122 |
| (>= 20.1) | 1.0000 | 0.4031 | 0.5969 | 0.5969 |
| (>= 24) | 0.6250 | 0.0357 | 0.9643 | 0.5893 |
| (>= 23.5) | 0.6250 | 0.0459 | 0.9541 | 0.5791 |
| (>= 23.3) | 0.6250 | 0.0510 | 0.9490 | 0.5740 |
| (>= 20) | 1.0000 | 0.5663 | 0.4337 | 0.4337 |
| (>= 25) | 0.4375 | 0.0051 | 0.9949 | 0.4324 |
| (>= 19.5) | 1.0000 | 0.5765 | 0.4235 | 0.4235 |
| (>= 24.5) | 0.4375 | 0.0153 | 0.9847 | 0.4222 |
| (>= 19.8) | 1.0000 | 0.7143 | 0.2857 | 0.2857 |
| (>= 18.5) | 1.0000 | 0.7245 | 0.2755 | 0.2755 |
| (>= 18.3) | 1.0000 | 0.7296 | 0.2704 | 0.2704 |
| (>= 18.2) | 1.0000 | 0.7398 | 0.2602 | 0.2602 |
| (>= 18.1) | 1.0000 | 0.7449 | 0.2551 | 0.2551 |
| (>= 26) | 0.2500 | 0.0051 | 0.9949 | 0.2449 |
| (>= 18) | 1.0000 | 0.8163 | 0.1837 | 0.1837 |
| (>= 17.9) | 1.0000 | 0.8265 | 0.1735 | 0.1735 |
| (>= 17.8) | 1.0000 | 0.8520 | 0.1480 | 0.1480 |
| (>= 17.7) | 1.0000 | 0.8571 | 0.1429 | 0.1429 |
| (>= 17.6) | 1.0000 | 0.8673 | 0.1327 | 0.1327 |
| (>= 27) | 0.1250 | 0.0000 | 1.0000 | 0.1250 |
| (>= 17.5) | 1.0000 | 0.8827 | 0.1173 | 0.1173 |
| (>= 17.1) | 1.0000 | 0.9031 | 0.0969 | 0.0969 |
| (>= 31) | 0.0625 | 0.0000 | 1.0000 | 0.0625 |
| (>= 17) | 1.0000 | 0.9490 | 0.0510 | 0.0510 |
| (>= 16.9) | 1.0000 | 0.9592 | 0.0408 | 0.0408 |
| (>= 16.5) | 1.0000 | 0.9847 | 0.0153 | 0.0153 |
| (>= 16.3) | 1.0000 | 0.9898 | 0.0102 | 0.0102 |
| (>= 16) | 1.0000 | 0.9949 | 0.0051 | 0.0051 |
| (>= 15.3) | 1.0000 | 1.0000 | 0.0000 | 0.0000 |
| (>= 31) | 0.0000 | 0.0000 | 1.0000 | 0.0000 |

Supplementary Table 2 d: A ROC analysis for determining the best optimal cut-off points MUAC in reference to MNA to screen malnutrition among older women in Borena District, South Wollo Zone, Ethiopia, March 2020 (n=212).

| Cut point | Sensitivity | 1-Specificity | Specificity | Youden Index |
| --- | --- | --- | --- | --- |
| (>= 30.1) | 0.9186 | 0.1066 | 0.8934 | 0.8120 |
| (>= 31.1) | 1.0000 | 0.2500 | 0.7500 | 0.7500 |
| (>= 30.7) | 1.0000 | 0.3010 | 0.6990 | 0.6990 |
| (>= 29.5) | 1.0000 | 0.3061 | 0.6939 | 0.6939 |
| (>= 29.2) | 1.0000 | 0.3112 | 0.6888 | 0.6888 |
| (>= 29.1) | 1.0000 | 0.3163 | 0.6837 | 0.6837 |
| (>= 32) | 0.6250 | 0.0561 | 0.9439 | 0.5689 |
| (>= 31.8) | 0.6250 | 0.0612 | 0.9388 | 0.5638 |
| (>= 29) | 1.0000 | 0.4490 | 0.5510 | 0.5510 |
| (>= 28.3) | 1.0000 | 0.4592 | 0.5408 | 0.5408 |
| (>= 31.2) | 0.6875 | 0.1480 | 0.8520 | 0.5395 |
| (>= 31.5) | 0.6250 | 0.0867 | 0.9133 | 0.5383 |
| (>= 31.24) | 0.6250 | 0.0918 | 0.9082 | 0.5332 |
| (>= 28) | 1.0000 | 0.5459 | 0.4541 | 0.4541 |
| (>= 27.2) | 1.0000 | 0.5510 | 0.4490 | 0.4490 |
| (>= 27.1) | 1.0000 | 0.5561 | 0.4439 | 0.4439 |
| (>= 32.1) | 0.4375 | 0.0102 | 0.9898 | 0.4273 |
| (>= 32.2) | 0.3750 | 0.0000 | 1.0000 | 0.3750 |
| (>= 27) | 1.0000 | 0.6531 | 0.3469 | 0.3469 |
| (>= 26.9) | 1.0000 | 0.6582 | 0.3418 | 0.3418 |
| (>= 26.5) | 1.0000 | 0.6735 | 0.3265 | 0.3265 |
| (>= 26.3) | 1.0000 | 0.6786 | 0.3214 | 0.3214 |
| (>= 26.2) | 1.0000 | 0.6837 | 0.3163 | 0.3163 |
| (>= 33) | 0.3125 | 0.0000 | 1.0000 | 0.3125 |
| (>= 26) | 1.0000 | 0.7347 | 0.2653 | 0.2653 |
| (>= 25.1) | 1.0000 | 0.7398 | 0.2602 | 0.2602 |
| (>= 34) | 0.2500 | 0.0000 | 1.0000 | 0.2500 |
| (>= 25) | 1.0000 | 0.7755 | 0.2245 | 0.2245 |
| (>= 24.5) | 1.0000 | 0.7908 | 0.2092 | 0.2092 |
| (>= 24.1) | 1.0000 | 0.8010 | 0.1990 | 0.1990 |
| (>= 35) | 0.1250 | 0.0000 | 1.0000 | 0.1250 |
| (>= 24) | 1.0000 | 0.8776 | 0.1224 | 0.1224 |
| (>= 23.8) | 1.0000 | 0.8827 | 0.1173 | 0.1173 |
| (>= 23.4) | 1.0000 | 0.8878 | 0.1122 | 0.1122 |
| (>= 23.2) | 1.0000 | 0.8929 | 0.1071 | 0.1071 |
| (>= 23.1) | 1.0000 | 0.8980 | 0.1020 | 0.1020 |
| (>= 39) | 0.0625 | 0.0000 | 1.0000 | 0.0625 |
| (>= 23) | 1.0000 | 0.9388 | 0.0612 | 0.0612 |
| (>= 22) | 1.0000 | 0.9796 | 0.0204 | 0.0204 |
| (>= 21.5) | 1.0000 | 0.9847 | 0.0153 | 0.0153 |
| (>= 21) | 1.0000 | 0.9949 | 0.0051 | 0.0051 |
| (>= 20) | 1.0000 | 1.0000 | 0.0000 | 0.0000 |
| (>= 39) | 0.0000 | 0.0000 | 1.0000 | 0.0000 |
